# Ubiquitin-Specific Protease Inhibitors for Cancer Therapy: Recent Advances and Future Prospects

**DOI:** 10.3390/biom15020240

**Published:** 2025-02-07

**Authors:** Mohamad Bakkar, Sara Khalil, Komal Bhayekar, Narva Deshwar Kushwaha, Amirreza Samarbakhsh, Sadaf Dorandish, Holly Edwards, Q. Ping Dou, Yubin Ge, Navnath S. Gavande

**Affiliations:** 1 Department of Pharmaceutical Sciences, Eugene Applebaum College of Pharmacy and Health Sciences (EACPHS), Wayne State University, Detroit, MI 48201, USA; mbakkar@med.wayne.edu (M.B.); hc2647komal@wayne.edu (K.B.); hn3752@wayne.edu (N.D.K.); gk1514@wayne.edu (A.S.); sdorandish@wayne.edu (S.D.); 2 Division of Pediatric Hematology and Oncology, Children’s Hospital of Michigan, Detroit, MI 48201, USA; 3 Cancer Biology Graduate Program, Wayne State University School of Medicine, Detroit, MI 48201, USA; ho9320@wayne.edu (S.K.); doup@karmanos.org (Q.P.D.); 4 Department of Oncology, Wayne State University School of Medicine, Detroit, MI 48201, USA; pitmanh@karmanos.org; 5 Molecular Therapeutics Program, Barbara Ann Karmanos Cancer Institute (KCI), Wayne State University School of Medicine, Detroit, MI 48201, USA

**Keywords:** ubiquitin-specific proteases (USPs), deubiquitinating enzymes (DUBs), small molecule inhibitors, cancer therapy

## Abstract

Cancer management has traditionally depended on chemotherapy as the mainstay of treatment; however, recent advancements in targeted therapies and immunotherapies have offered new options. Ubiquitin-specific proteases (USPs) have emerged as promising therapeutic targets in cancer treatment due to their crucial roles in regulating protein homeostasis and various essential cellular processes. This review covers the following: (1) the structural and functional characteristics of USPs, highlighting their involvement in key cancer-related pathways, and (2) the discovery, chemical structures, mechanisms of action, and potential clinical implications of USP inhibitors in cancer therapy. Particular attention is given to the role of USP inhibitors in enhancing cancer immunotherapy, e.g., modulation of the tumor microenvironment, effect on regulatory T cell function, and influence on immune checkpoint pathways. Furthermore, this review summarizes the current progress and challenges of clinical trials involving USP inhibitors as cancer therapy. We also discuss the complexities of achieving target selectivity, the ongoing efforts to develop more specific and potent USP inhibitors, and the potential of USP inhibitors to overcome drug resistance and synergize with existing cancer treatments. We finally provide a perspective on future directions in targeting USPs, including the potential for personalized medicine based on specific gene mutations, underscoring their significant potential for enhancing cancer treatment. By elucidating their mechanisms of action, clinical progress, and potential future applications, we hope that this review could serve as a useful resource for both basic scientists and clinicians in the field of cancer therapeutics.

## 1. Introduction

### 1.1. Overview of the Ubiquitin–Proteasome Pathway

**Figure 1:**
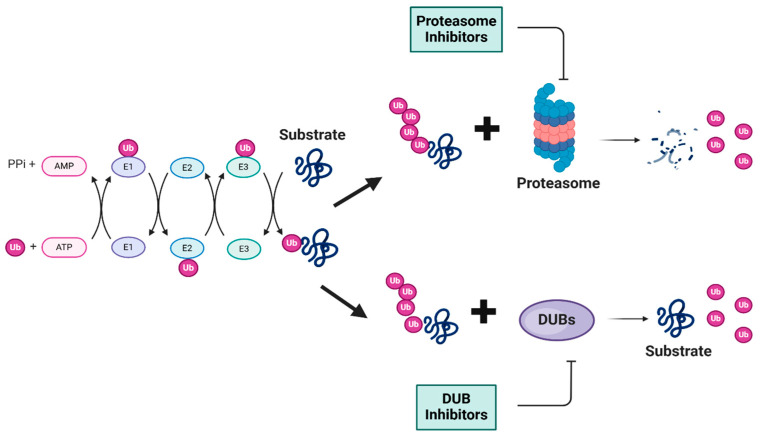
Schematic representation of ubiquitin-tagged protein degradation. This schematic illustrates the UPS, a vital cellular protein degradation mechanism. The process begins with the ATP-dependent activation of ubiquitin, which leads to a cascade of enzymatic reactions that ultimately results in the degradation of the substrates into smaller peptides by the proteasome or stabilization of the substrates by the relevant DUB. In both cases, ubiquitin molecules are released for reuse. Ub: ubiquitin (Ub) molecules (purple); ATP/AMP: energy source for ubiquitin activation; PPi: inorganic pyrophosphate; proteasome: multi-subunit protein degradation complex (blue and pink); degradation products: released peptide fragments and free ubiquitin molecules; DUBs: deubiquitinating enzymes. (Created using BioRender. Bakkar, M. (2025)).

The ubiquitin–proteasome system (UPS) is a vital pathway regulating protein levels in eukaryotic cells, which is essential for maintaining cellular homeostasis. This pathway consists of several enzymatic steps that ensure the targeted degradation of substrate proteins. The UPS process begins with the activation of ubiquitin (Ub) by the E1 ubiquitin-activating enzyme in an ATP-dependent reaction, which is then transferred to an E2 ubiquitin-conjugating enzyme and finally transferred to a specific lysine residue on the target proteins by an E3 ubiquitin ligase (Figure 1). This process is repeated to form a polyubiquitin chain on the target proteins, which serves as a signal for recognition by the 26S proteasome [1]. The 26S proteasome, a multi-subunit protease complex consisting of a 20S core particle with proteolytic activity and two 19S regulatory particles that recognize ubiquitinated proteins, is then responsible for the degradation of the targeted substrate proteins into small peptides. Deubiquitinating enzymes (DUBs) play a significant role in the regulation of the UPS by removing ubiquitin molecules from ubiquitinated substrate proteins. The UPS is involved in multiple cellular processes, including DNA damage response (DDR), apoptosis, signal transduction, and drug resistance. Dysfunction of the UPS has been implicated in a range of disease types, including cancer [2]. Cancers with higher protein turnover are generally more sensitive to the inhibition of proteasome and deubiquitinases. The importance of the UPS in pathogenesis is underscored by the development of proteasome inhibitors as therapeutic agents. To date, the FDA has approved three 20S proteasome inhibitors, Borezomib, Carfilzomib, and Ixazomib, for the treatment of hematologic malignancies, namely multiple myeloma and mantle cell lymphoma. Bortezomib, which was discovered in 1995 and approved by the US FDA for the treatment of multiple myeloma in 2003 and later for the treatment of mantle cell lymphoma, validated the therapeutic potential of targeting the UPS, particularly in hematologic malignancies [3]. Carfilzomib was subsequently approved in 2012, followed by Ixazomib, both specifically for the treatment of multiple myeloma. These drugs have offered new therapeutic options for patients with these challenging blood cancers [4]. The UPS’s roles in a wide range of cellular processes make it an important target of ongoing research.

### 1.2. Role of Deubiquitinating Enzymes

Deubiquitinating enzymes (DUBs) are proteases that cleave ubiquitin from various substrate proteins, playing a crucial role in regulating the ubiquitin signaling pathway. The human genome encodes approximately 100 putative DUBs, which are classified into two main classes: metalloproteases and cysteine (Cys) proteases. These classes are further subclassed based on their domain structures. The first family belongs to the JAB1/MPN/Mov34 metalloenzyme (JAMM) domain zinc-dependent metalloprotease family, while the other five families—the ubiquitin C-terminal hydrolases (UCH), the ovarian tumor proteases (OTU), the Machado–Josephin domain proteases (MJDs), the ubiquitin-specific protease (USP/UBP), and the recently discovered motif interacting with ubiquitin (MIU)-containing novel DUB family (MINDY) and Zinc finger with UFM1-specific peptidase domain protein (ZUFSP)—are papain-like cysteine proteases.

#### 1.2.1. Metalloprotease DUBs

Metalloproteases form one of the two main classes of DUBs, with the primary subclass of metalloproteases being JAMM (JAB1/MPN/MOV34 metalloenzyme) domain proteases. The catalytic site of this subclass of DUBs contains two histidine residues, an aspartate (Asp), and a catalytic serine (Ser). The main unique property of metalloproteases is that they use zinc in their catalytic mechanism, which distinguishes them from the cysteine protease DUBs. The zinc ion plays a role in the generation of a hydroxide ion from water, which acts as a nucleophile to hydrolyze the isopeptide bond between the protein substrate and ubiquitin. A key consequence of this mechanism is that this type of DUB does not form a covalent intermediate with the substrate during catalysis. In contrast to ubiquitin-specific proteases (USPs), which use their catalytic serine to form a covalent intermediate with the substrate during enzyme cleavage, metalloprotease DUBs utilize a nucleophilic hydroxide ion that directly attacks the peptide bond without forming a covalent bond with the enzyme. This characteristic makes them inherently resistant to DUB inhibitors, which often target the formation of such covalent intermediates [5].

#### 1.2.2. Cysteine Protease DUBs

The six subfamilies of cysteine protease DUBs are organized based on their domain architecture. It is important to note that most DUBs in this class utilize a catalytic triad composed of a histidine (His), an active site cysteine (Cys), and in most cases, an asparagine (Asn) or aspartate (Asp).

##### Ubiquitin C-Terminal Hydrolases (UCHs)

This subfamily of cysteine proteases was one of the first types of DUBs identified with UCHL3 discovered in 1997 [6]. The UCH domain contains three conserved residues, namely His, Cys, and Asp, and there are four known members of this subfamily. These small enzymes preferentially remove small peptides from the C-terminus of ubiquitin. The loop structure covering the active site limits the size of the substrate with which they can interact, unlike USPs, which can handle larger substrates. UCHs have been implicated in playing a role in the oncogenesis of various cancers [7].

##### Ovarian Tumor Proteases (OTUs)

This subfamily displays specificity for the ubiquitin substrates with which it interacts by utilizing additional ubiquitin interaction sites that can bind to specific linkages on longer protein chains. Another important distinction is the lack of an asparagine or aspartate residue in some members of this subfamily [5]. OTUD5 is an important member of the OTU subfamily of cysteine proteases and has been implicated in both tumor progression and tumor suppression depending on the disease type. These DUBs play an important role in various cellular processes, including DNA repair and protein quality control [8].

##### Machado–Josephin Domain Proteases (MJDs)

These DUBs comprise four members in humans, and all contain a catalytic domain known as the Josephin domain. This important feature contains two conserved histidines, a catalytic cysteine, and two ubiquitin-binding sites. This subfamily is named after the Machado–Josephin disease, a neurological disorder caused by a CAG repeat expansion motif producing polyglutamine resulting in protein aggregation and misfolding [9]. Ataxin-3, a critical MJD in humans, has been shown to play a role in the proliferation of testicular cancer, gastric cancer, and lung cancer cells [10].

##### Motif Interacting with Ubiquitin (MIU)-Containing Novel DUB Family (MINDY) and Zinc Finger with UFM1-Specific Peptidase Domain Protein (ZUFSP)

These two subfamilies of cysteine proteases were the two most recently identified. The MINDY subfamily of DUBs has a unique catalytic triad consisting of cysteine, histidine, and threonine [11]. There are four known members of this subfamily, and these enzymes appear to preferentially cleave long ubiquitin chains starting from the direction of the distal end [12]. ZUFSP contains only one known member in humans, which utilizes multiple ubiquitin-binding domains and possesses highly specific cleavage of K63 ubiquitin linkage [13]. ZUFSP has a modular architecture, with its overall specificity and activity being influenced by its various modular ubiquitin-binding domains [14].

##### Ubiquitin-Specific Proteases (USPs)

USPs form the largest subclass of DUBs with 58 known members and are the focus of this review article. USPs are generally larger enzymes, ranging from 50 to 300 kDa and typically have N-terminal extensions involved in substrate recognition and protein–protein interactions. USPs can remove ubiquitin from protein conjugates, process ubiquitin precursors, and disassemble ubiquitin chains. Some types of USPs show specificity for certain substrates, while others have broader activity. They play crucial roles in various cellular processes including the cell cycle, protein degradation, signal transduction, and DNA repair. Their substrate specificities make them important potential targets for therapeutic development in the treatment of diseases such as cancer [5]. Given the crucial role USPs play in regulating protein homeostasis as well as their potential as therapeutic targets in cancer therapy, the remainder of this review will focus on the structural and functional characteristics of USPs, their involvement in key cancer-related pathways, and the development of USP inhibitors as promising anticancer agents.

## 2. USPs: Structure, Biological Function, and Role in Tumorigenesis and Cancer Progression

### 2.1. The Structure of USPs

USPs represent the most prominent family of DUBs. They represent a sophisticated class of enzymes whose function depends on the intricate interplay between three vital structural elements. These elements—the core catalytic domain, ubiquitin-like domains, and ancillary domains—work congruently to achieve precise protein regulation [15]. Their activities are fine-tuned through post-translational modifications (PTMs), creating a complex regulatory network [16]. Understanding this structural and regulatory framework is crucial for basic research and therapeutic development. As common with signaling proteins, most USP deubiquitinases have a modular architecture with a catalytic domain, putative site of interaction, and localization domains.

Most USP domains have at least two ubiquitin-binding sites, one for the proximal ubiquitin molecules and one for the distal ubiquitin molecules. These sites cleave the isopeptide bond linking two ubiquitin moieties or the proximal ubiquitin with a protein substrate [17]. The USP family is the largest, with more than 50 members. The USP catalytic domain may be flanked at its N- or C-terminus by several additional domains involved in differential substrate recognition, sub-cellular localization, and regulation of enzymatic activity [18]. Among the members of the USP domain family, the majority act upon larger protein substrates in contrast to other subclasses of DUBs [19].

#### 2.1.1. Core Catalytic Domain

**Figure 2:**
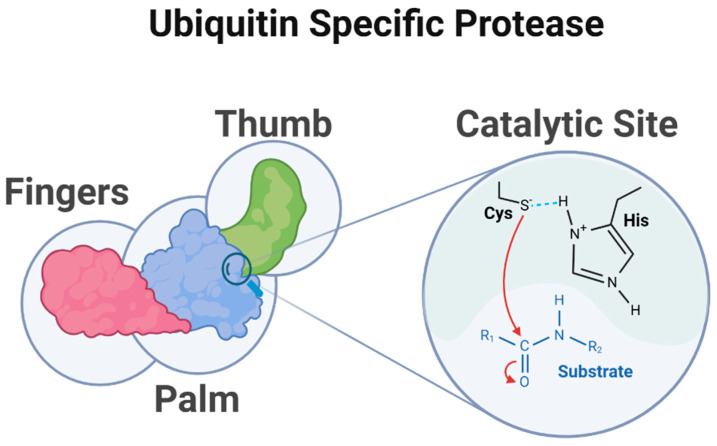
Structure of ubiquitin-specific protease. This diagram depicts the structural organization and catalytic mechanism of a USP. The USP contains a catalytic core domain featuring the characteristic catalytic triad consisting of cysteine (Cys), histidine (His), and, in most cases, an asparagine (Asn) or aspartate (Asp), which interacts with histidine (the cysteine and histidine residues are illustrated in the figure) highlighted in the active site between the palm and thumb sub-domains. The isopeptide bond between ubiquitin and the substrate protein is cleaved resulting in the removal of ubiquitin modifications, which is crucial for protein regulation and cellular homeostasis. (Created using BioRender. Bakkar, M. (2025)).

At the heart of USP function is the core catalytic domain, whose structure reveals the precise ingenuity of these enzymes. This domain features homolog sequences derived from papain-like cysteine proteases arranged in a distinctive “palm, thumb, and fingers” configuration (Figure 2) that orchestrates substrate binding and cleavage [20,21].

**Figure 3:**
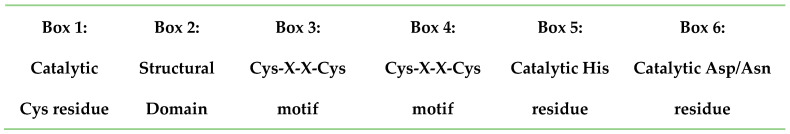
Molecular basis of ubiquitin-specific protease catalytic core protein domains.

The catalytic center of USPs is located at the interface between the palm and thumb regions of the USP domain. The USP catalytic core can be thought of as being divided into six conserved boxes (Figure 3).

Box 1 includes a catalytic Cys residue, box 5 includes a catalytic His, and box 6 includes a catalytic Asp/Asn residue. Each of these boxes is characterized by several other conserved features and residues. Boxes 3 and 4 contain a Cys-X-X-Cys motif, which has been reported to form a functional zinc-binding motif in some USPs. It is speculated that zinc binding facilitates the folding of the USP core, thus allowing the interaction of sequence motifs that are several hundred residues apart. USP domains exhibit a common conserved fold. The catalytic triad resides between the thumb (Cys) and palm sub-domains (His/Asp) [17]. The principal site of the DUB has a strong interaction with the distal ubiquitin, mainly through the Ile44 patch, with different interacting surfaces within DUB subfamilies. The stretch of amino acids extending from the binding site of the C-terminus of the distal ubiquitin to the DUB catalytic center is responsible for distinguishing ubiquitin from other ubiquitin-like molecules (ULMs) [22,23]. The residues that are responsible for the difference in the C-terminal sequence of ubiquitin (Leu71, Arg72, Leu73, Arg74, Gly75, Gly76) when compared to that of ULMs are vital for identifying ubiquitin by DUBs [22,24]. The catalytic domain is responsible for the isopeptidase activity that cleaves the isopeptide bond between ubiquitin and the target substrate protein [18]. Most USPs contain a core catalytic domain with insertions and terminal extensions bearing additional protein interaction domains [20].

#### 2.1.2. Ubiquitin-like Domains (UBL)

Building upon this catalytic foundation, UBL domains extend the functional repertoire of USPs. Strategically positioned near the distal ubiquitin-binding site, they play a crucial role in substrate recognition and regulation. Their integration within the catalytic core’s variable insertions facilitates essential intra-molecular interactions that maintain proper USP conformation. Many USPs contain UBL domains that can bind to ubiquitin or other UBLs, as well as compete with ubiquitin binding and regulate catalysis. These UBL domains may mediate substrate recognition and binding, as well as play a vital role in localization at the proteasome [18].

#### 2.1.3. Ancillary Domains

Complementing these two core components mentioned above, ancillary domains add another layer of functional sophistication through specialized structural elements. These include zinc finger domains, ubiquitin-interacting motifs (UIMs), and SUMO-interacting motifs (SIMs) that regulate their activity, localization, and substrate specificity [18]. These domains have fine-tuned substrate specificity and mediate crucial protein–protein interactions, although the role of these ancillary domains in regulating USP activity is not always consistent [21]. Their structural adaptability, particularly in the substrate-induced ordering of the unstructured region, provides additional regulatory flexibility through PTM-mediated control [17].

### 2.2. The Roles of USPs in Tumorigenesis and Cancer Progression

USPs regulate several critical cancer-related pathways, including NF-κB, Wnt/β-catenin, JAK/STAT, p53 signaling, c-MYC, TGF-β, DNA repair, apoptosis, cell cycle regulation, MAPK, and hypoxia pathways (Table 1). These pathways are involved in various aspects of cancer biology, such as cellular proliferation, survival, metastasis, and therapy resistance. USPs modulate these pathways through deubiquitination of key substrate proteins, which affects their stability and, consequently, their functions. This regulation can either promote or inhibit cancer progression depending on the specific USP and pathway involved. The diverse roles of USPs in these pathways highlight their importance in cancer biology and their potential as therapeutic targets.

### 2.3. Unique Structural and Functional Characteristics of Various USPs

USPs are a diverse family of DUBs that play crucial roles in protein homeostasis. A common theme among many USPs is their association with cancer pathogenesis, highlighting the importance of ubiquitin regulation in malignancies. USPs share some common structural features, but they also possess unique elements that contribute to their specific functions [53]. Table 2 provides a description of some of the similarities and differences of USPs.

The mechanism of action of the USP family of DUBs is generally conserved across members. The enzymatic activity relies on nucleophilic substitution of the catalytic cysteine on the isopeptide bond between ubiquitin and its protein substrate. The histidine residue acts as a general base and activates the catalytic cysteine for this reaction by lowering its pKa (shown in Figure 2). This mechanism is crucial for the deubiquitinating activity of USPs, allowing them to remove ubiquitin modifications from target proteins and thereby regulate their stability and functions [18].

Beyond the catalytic domain, USPs often have additional structural elements that regulate their activity and confer specificity. These include ubiquitin-binding domains, zinc finger domains, and other protein–protein interaction motifs that allow USPs to recognize specific substrates or participate in particular cellular pathways [18]. Despite their structural and functional similarities, the development of specific inhibitors for USPs has been uneven. Several USP inhibitors have been developed through various drug discovery approaches, including high-throughput screening and structure-based drug design. However, for many USPs, specific inhibitors have not yet been discovered [54]. This gap represents both a challenge and an opportunity in the field of cancer drug development and research, as targeting specific USPs could lead to novel cancer therapies that can potentially overcome drug resistance, improve efficacy, and reduce side effects.

**Table 2:** Unique structural and functional characteristics of various USPs.

USP Name	Domains	Catalytic Site	Cancer Association	References
USP1	Conserved USP catalytic domain (thumb, palm, and finger sub-domains).	Three catalytic residues: Cys90, His593, and Asp751.	Osteosarcoma, renal clear cell carcinoma, colorectal carcinoma, non-small cell lung cancer, and gastric cancers	[42]
USP3	Zinc finger (ZnF) domain and catalytic domain.	Conserved catalytic domain containing key Cys, Hys, and Asp/Asn residues critical for USP activity.	Gastric cancer and breast cancer	[55,56]
USP5	Catalytic domain flanked by two ubiquitin-associated (UBA) domains and two Zinc finger (ZnF) ubiquitin-binding protein (UBP) domains.	Papain-like proteases have a Cys and a His residue, which form an ion pair with the negatively charged cysteine thiolate functioning as a nucleophile.	Breast, lung, colorectal, hepatocellular, pancreatic, and non-small cell lung cancer	[57,58,59]
USP7	N-terminal MATH domain, central catalytic domain, and five C-terminal tandem ubiquitin-like domains (UbL).	Cys223, His464, and Asp481 residues are critical for deubiquitination.	Colorectal cancer, osteosarcoma, acute myeloid leukemia, breast cancer, prostate cancer, multiple myeloma, ovarian cancer, bladder cancer, esophageal squamous cell carcinoma, chronic lymphocytic lymphoma, and medulloblastoma	[54,60,61]
USP10	N-terminus region, USP catalytic structure domain, and a smaller C-terminus region.	Conserved catalytic domain containing key Cys, His, and Asp/Asn residues critical for USP activity.	Colorectal cancer, prostate cancer, hepatocellular carcinoma, glioblastoma multiforme, non-small-cell lung cancer, chronic myeloid leukemia, and acute myeloid leukemia	[62]
USP19	Two CHORD-SGT1/P23 domains (CS1 and CS2 at the N-terminus), USP catalytic domain.	Contains Cys and His residues critical for enzymatic activity and auto-inhibition mechanism.	Breast cancer and osteosarcoma	[63,64]
USP20	ZnF-UBP domain, catalytic USP domain, and two domains present in ubiquitin-specific protease (DUSP) domains.	Conserved Cys and His residues that catalyze proteolysis of the isopeptide bond between a target protein lysine residue and a glycine residue of ubiquitin.	Breast cancer, lung cancer, colon cancer, gastric cancer, and adult T-cell leukemia	[44,65]
USP32	USP catalytic domain, Domain in USP (DUSP), two UbL domains, and calcium-binding EF-hand with a signal transduction mechanism.	Conserved catalytic domain containing key Cys and His residues critical for USP activity.	Small cell lung cancer, gastric cancer, breast cancer, epithelial ovarian cancer, glioblastoma, gastrointestinal stromal tumor, AML, and pancreatic adenocarcinoma	[66]
USP36	Conserved USP catalytic domain (thumb, palm, and finger sub-domains).	Key residues: Cys223, His464, Asp481 in the catalytic domain.	Colon cancer and breast cancer	[36,67]

### 2.4. History of the Development of USP Inhibitors

Over the past decade, the development of USP inhibitors has gained significant momentum as a promising area in cancer therapeutics. Discovery of USP inhibitors primarily relied on high-throughput screening prior to 2014. However, the field has since evolved with structure-guided drug design based on co-crystal structure complexes becoming a prominent approach [68,69]. The interest in USP inhibitors stems from the crucial role USPs play in various cellular processes and their involvement in multiple cancer-related pathways [54]. The shift in focus to targeting USPs was partly due to the limitations of existing UPS-targeted inhibitors such as Bortezomib, which showed efficacy primarily in a couple of hematologic malignancies but only marginal effects on solid tumors [68]. More than seventy USP inhibitors have been reported over the past 20 years with six reaching clinical trials, but despite these advancements, no USP inhibitor has yet been approved for clinical use [69]. The development of USP inhibitors faces the challenge of achieving target selectivity, but despite this, there is growing optimism that USPs represent a new reservoir of therapeutic targets [54].

### 2.5. Therapeutic Implications

The therapeutic significance of the USP structural organization becomes apparent in disease contexts, particularly in cancer biology. This integrated understanding of USP structure and regulation reveals a remarkable system where each domain contributes to a more extensive functional network. PTMs modulate the activity of signaling proteins further, forming an intricate regulatory network evident in several disease processes, particularly in cancer biology. Specifically, these modifications regulate essential cellular processes, including proliferation, immune responses, and apoptosis [70]. Drug discovery efforts over the last decade have capitalized on this knowledge for the development of adequate targeted therapeutic strategies.

## 3. Small Molecule Inhibitors Targeting USPs

### 3.1. Chemical Structure, Inhibition Potency, and Mechanism of Action of Small Molecule Inhibitors Targeting USPs

To date, more than seventy small-molecule USP inhibitors have been reported in the literature. The chemical structures, inhibition potency, and mechanisms of action of some selected USP inhibitors are summarized in Table 3. USP inhibitors that are currently being or were previously tested in cancer clinical trials will be discussed in Section 3.3. USP inhibitors can be broadly categorized based on their specificity and the USPs they target. Some USP inhibitors, such as SP-002 and Pimozide (PMZ), are highly selective for USP1, while others like PR619 are pan-inhibitors affecting multiple USPs. The mechanisms of action vary among these compounds, with some binding reversibly to their targets (e.g., IU1), while others form irreversible covalent bonds (e.g., Q29, XL177A). Many of these inhibitors demonstrate promising anticancer effects through various mechanisms. Common themes include inducing cell cycle arrest, enhancing DNA damage, promoting apoptosis, and modulating key signaling pathways, as described in Table 1. For instance, USP7 inhibitors HBX-19818 and P22077 work by stabilizing p53 and promoting the degradation of oncogenic proteins. USP14 inhibitors like IU1 and its derivatives have shown potential in treating neurodegenerative disorders by modulating the degradation of tau protein. Research on these USP inhibitors spans various stages, from in vitro studies to in vivo animal models, with some compounds showing efficacy in xenograft mouse models. Several inhibitors have been shown to exhibit synergistic effects when combined with other cancer therapies, such as PARP inhibitors or traditional chemotherapeutic agents. While these USP inhibitors show promise as a novel class of anticancer drugs in preclinical studies, the potential of these agents in clinical settings needs to be determined.

### 3.2. USP Inhibitors and Immunotherapy

As mentioned above, the development of USP inhibitors represents a significant advancement in cancer treatment, offering a multifaceted approach to improving patient outcomes. USP inhibitors work by various mechanisms to inhibit cancer cell growth and proliferation, such as destabilizing oncoproteins [181]. USP inhibitors thus offer a novel strategy to overcome drug resistance, a major challenge in cancer therapy, by targeting the mechanisms that cancer cells employ to evade chemotherapy and targeted treatments [182]. Some USP inhibitors also show promise in enhancing cancer immunotherapy by making cancer cells more vulnerable to immune attacks [183]. In this section, we focus on the potential of USP inhibitors in enhancing the immune system and immunotherapy against cancer.

#### 3.2.1. Role of USP1 in Immune Response

USP1 plays a significant role in immunotherapy, particularly in the context of cancer treatment. USP1 has been identified as a critical regulator of T-cell differentiation, which is crucial for effective immunotherapy responses. By deubiquitinating and stabilizing the transcriptional co-activator with PDZ-binding motif (TAZ), USP1 leads to enhanced activity of RORγt, which is an important transcription factor for T helper type 17 (Th17) cell development. Deubiquitination of TAZ also causes decreased acetylation of Foxp3, which promotes its proteasomal degradation leading to reduced Treg cell differentiation [184]. USP1 has also been implicated in resistance to chemotherapy and Rituximab in DLBCL. USP1 deubiquitinates MAX, which is an important MYC-binding protein and promotes MYC gene transcription. High expression of USP1 in DLBCL has been found to be associated with a poorer prognosis, while inhibition with Pimozide or knockdown of USP1 has been shown to reduce cancer cell growth and induce cell cycle arrest. Inhibition of USP1 was also demonstrated to significantly reduce tumor burden in a therapy-resistant DLBCL-engrafted PDX mouse model. Importantly, Pimozide was shown to have evidence of synergy with Etoposide in therapy-resistant DLBCL [80]. The potential of USP1 inhibitors lies in their ability to modulate the tumor microenvironment and enhance the efficacy of immunotherapeutics. Combining USP1 inhibitors with existing immunotherapies may enhance treatment efficacy, particularly in cases of resistance to current therapies.

#### 3.2.2. Role of USP7 in Immune Response

Recent research has substantially advanced our understanding of how USP7 modulates the immune response in cancer patients. Elevated levels of USP7 have been shown to facilitate tumor growth by enhancing the immunosuppressive functions of Foxp3+ Tregs [185,186]. Blocking USP7 hinders the activity of Tip60-dependent Foxp3+ Treg cells, decreasing their inhibitory function and enhancing the body’s ability to fight against tumors [186]. Histone acetyltransferase Tat-interactive protein (Tip60) is essential for the survival of Treg cells. Tip60 is a USP7 substrate, thus targeting USP7 could interfere with Tip60-mediated Foxp3 dimer formation, resulting in reduced activation of immunosuppressive molecules such as CTLA4 and IL-10 while promoting the expression of pro-inflammatory cytokines such as IL-2 and IFN-γ [186]. USP7 is important in regulating the equilibrium of M1 (which suppresses tumor growth and promotes an anti-tumor immune response) and M2 (which aids in tumor progression and inhibits the immune system) macrophages. Targeted inhibition of USP7 results in a change in the appearance and behavior of M2 macrophages, leading to a higher growth rate of differentiated CD8+ T cell groups in a laboratory setting [61]. In research on mice with Lewis lung carcinoma, inhibiting USP7 led to decreased tumor growth and increased levels of M1 macrophages and CD8+ T cells that expressed IFN-γ. USP7 also maintains the stability of PD-L1 by inhibiting its degradation [187].

#### 3.2.3. Enhancing Immunotherapy by USP Inhibitors

USP inhibitors can make cancer cells more susceptible to immune attack by preventing them from evading the immune system and enhancing the effectiveness of immunotherapy [183]. Tumors have specific properties that allow them to evade the immune system, hindering an anti-tumor response and promoting their growth. Mechanisms of immune evasion include the production of immunosuppressive factors, down-regulation of major histocompatibility complex molecules, and recruitment of immunoregulatory cells. Additional studies have shown that USPs can influence the effectiveness of immunotherapy through the regulation of immune cell function and the immune response within the tumor microenvironment [157,188].

HBX-19818 and GNE-6776 are covalent USP7 inhibitors that have shown promise in stabilizing p53 and enhancing its activity, which can lead to improved anti-tumor responses [116]. USP22 inhibitors have also shown potential in improving responses to immunotherapy by increasing levels of CD8+ T cells and NK cells, transforming immune-desert tumors into immune-inflamed tumors [189]. In liver tumors, inhibiting USP22 has been shown to boost tumor immunogenicity, enhance T-cell infiltration, and increase responsiveness to anti-PD-L1 immunotherapy [183,190]. Recent research has identified potent macrocycle inhibitors of USP22, including compound S02, which binds tightly to the catalytic domain pocket of USP22. These USP22 inhibitors have demonstrated significant potential in reducing cancer cell growth, promoting apoptosis, and enhancing the efficacy of existing treatments [183]. USP8 inhibitors have shown potential in enhancing anti-tumor activity when combined with anti-PD-1/PD-L1 immunotherapy. USP13 inhibitors may enhance the antitumor effects of DNA damage response inhibitors as well as promote immune cell infiltration and innate immunity [191]. USP14 inhibitors have been shown to sensitize cancer cells to immune checkpoint inhibitors [192]. USP15 inhibitors can disrupt pathways controlling Toll-like receptor, RIG-I, and NF-kB signaling, enhancing the immune response against tumors [183]. USP9X inhibitors like G9 have shown promise in inactivating Notch signaling, reducing proinflammatory cytokines, and enhancing antitumor immune response [183,193].

In summary, USP inhibitors represent a promising avenue for enhancing cancer immunotherapy by modulating various aspects of the immune response as well as the tumor microenvironment. Their ability to target multiple pathways involved in immune evasion and tumor progression and to enhance the efficacy of existing treatments makes them a valuable addition to the arsenal of cancer therapeutics.

### 3.3. Development of USP Inhibitors for Cancer Therapy: Clinical Trials Status

Regarding the development of USP inhibitors for the treatment of cancer, most of these inhibitors are still in the preclinical stage. However, a few USP inhibitors are being explored in clinical trials for their potential therapeutic effects, given their mechanism of action and possible benefits to patients with resistant or refractory disease. Multiple USP1 inhibitors are currently being tested in phase 1 and phase 2 clinical trials, which aim to evaluate the safety, tolerability, and potential antitumor activity of these compounds. While there are no USP7 inhibitors currently in clinical trials, several compounds have shown promise in preclinical studies. Inhibition of USP7 has long been viewed as a promising anticancer target being the key DUB in regulating p53 levels. FT671 and FT827 are both small-molecule USP7 inhibitors that have shown high affinity and selectivity to USP7 in vitro. Both compounds appear to target the auto-inhibited apo form of USP7 near the catalytic center, which is distinct from other USPs [123]. Most recently, YCH2823 was discovered as a next-generation USP7 inhibitor with enhanced cellular activity compared to FT671, demonstrating about five times the potency. YCH2823 inhibited the growth of *TP53*-wild-type, *TP53*-mutant, and *MYCN*-amplified cell lines with exceptional efficacy by binding directly to the catalytic domain of USP7. A synergy between USP7 and mTOR inhibitors was also observed, demonstrating potential for novel therapeutic strategies [126]. Table 4 provides a comprehensive overview of the USP inhibitors that are currently or were previously investigated in clinical trials. The study design, preliminary/current results, and key findings from these clinical studies are also described where applicable in the table.

#### 3.3.1. USP1 Inhibitors

USP1 inhibitors represent a promising class of drugs targeting DNA damage repair mechanisms, particularly in homologous recombination deficient (HRD) cancers. Several USP1 inhibitor compounds are currently being studied in various stages of clinical development. TNG348, developed by Tango Therapeutics, is an oral, potent, and highly selective allosteric USP1 inhibitor. It showed potential in preclinical models, demonstrating enhanced activity when combined with DNA repair pathway targeted therapies and efficacy in xenografts with both primary and acquired resistance to PARP inhibitors. Malignancies with HRD are generally more sensitive to therapies that target DNA repair. BRCA1-mutated tumor cells are known to be sensitive to PARP inhibitors due to defects in replication fork stability, and they have shown synergy with chemotherapy. HRD cancers represent up to 1 in 2 ovarian cancers, 1 in 4 breast cancers, 1 in 10 prostate cancers, and 1 in 20 pancreatic cancers. USP1 has been found to be upregulated in tumors with BRCA1 mutations, and its knockdown destabilizes replication forks resulting in cell death [194]. Inhibiting USP1 could be a potential therapeutic strategy for cancers with BRCA1-deficient cells, especially those resistant to PARP inhibitors [194]. However, the phase 1/2 trial (NCT06065059) was terminated due to significant liver toxicity, with grade 3/4 hepatotoxicity observed in patients who continued treatment for longer than eight weeks. These results demonstrate the challenges in developing novel therapies and highlight the importance of safety monitoring in early-phase clinical trials.

KSQ-4279, also known as RO7623066, was developed by Roche/KSQ Therapeutics, and it is a potent and highly selective oral USP1 inhibitor. It binds to a cryptic site in USP1, similar to ML323, but with subtle rearrangements in the site folding, accounting for differences in potency and selectivity [195]. Currently, in a phase 1 trial (NCT05240898) for advanced solid tumors, KSQ-4279 is being studied as a single agent and in combination with Carboplatin or Olaparib. The combination of a PARP inhibitor and KSQ-4279 was shown to induce regression of several PDX PARP-resistant tumors in the preclinical phase [196]. Preliminary results showed limited efficacy as monotherapy but suggested potential synergy with Olaparib, particularly in BRCA1-mutated tumors. Pharmacokinetic studies demonstrated that the tested drug becomes saturated at higher doses, suggesting the need for twice-daily dosing. Pharmacodynamic data also appeared to support the theorized mechanism of action of USP1 inhibition.

XL309, also known as ISM3091, was developed using AI technology by Insilico Medicine, Boston, Massachusetts, USA and licensed to Exelixis, Alameda, California, USA, and it is a highly selective and non-covalent oral inhibitor of USP1 [197]. Preclinical data demonstrated high efficacy in BRCA1 mutated triple-negative breast cancer cell lines and synergistic effects with Olaparib in vivo. Interestingly, it also showed efficacy against a lung adenocarcinoma cell line without HRD, suggesting potential applications beyond HRD cancers. This opens the door to consider potentially utilizing XL309 in HR-proficient cancers. A phase 1 trial (NCT05932862) is ongoing to investigate its safety and preliminary antitumor activity.

SIM0501, developed by Simcere Jiangsu Pharmaceutical Co., Nanjing, Jiangsu, China, is another highly selective and non-covalent oral USP1 inhibitor [195]. It has shown evidence of synergy with Olaparib both in vitro and in vivo across various HRD-positive cancers. In vivo efficacy studies have demonstrated a dose-dependent increase of Ub-PCNA, which may be a useful biomarker to track for response, as Ub-PCNA is a direct target of USP1 [196]. A phase 1 trial (NCT06331559) is evaluating its safety and preliminary efficacy in patients with advanced solid tumors.

HSK39775, developed by Tibet Haisco Pharmaceutical Co. Ltd., Zedang Town, Shannan, Tibet, is a highly selective and non-covalent oral USP1 inhibitor. It has been shown to inhibit the USP1/UAF1 complex and exhibit strong dose-dependent tumor response in xenograft-derived BRCA-mutated triple-negative breast cancer with synergy seen when given with a PARP inhibitor. HSK39775 monotherapy was also found to inhibit the growth of BRCA wild-type lung cancer [196]. A phase 1/2 trial (NCT06314373) is underway to evaluate its safety and efficacy in advanced solid tumors. The field of USP1 inhibitors is rapidly evolving, and these clinical trials will provide crucial data for this novel therapeutic approach in cancer management, particularly for patients with HRD-positive tumors and those resistant to PARP inhibitors.

#### 3.3.2. USP14 Inhibitor

VLX1570, a small molecule DUB inhibitor derived from b-AP15 [(3E,5E)-3,5-bis[(4-nitrophenyl)methylidene]-1-(prop-2-enoyl)piperidin-4-one], selectively inhibits USP14 and UCHL5, which are associated with the 19S regulatory subunit of the proteasome. Both of these small molecule inhibitors are α,β-unsaturated carbonyl DUB inhibitors and have similar mechanisms of action. It was studied in a phase 1 clinical trial (NCT02372240) for relapsed or refractory multiple myeloma patients who had developed resistance to Bortezomib [198]. The trial, conducted by Eric K Rowinsky et al., aimed to determine the safety and tolerability of VLX1570. While the compound showed anti-myeloma effects at doses ≥ 0.6 mg/kg, the study was discontinued due to severe respiratory insufficiency in two patients that led to death [199]. Despite these setbacks, the unique mechanism of action of DUB inhibitors in Bortezomib-resistant multiple myeloma has prompted further research, including the development of a rat model to assess VLX1570-induced lung toxicity and the development of specific DUB inhibitors [199].

**Table 4:** Clinical trials involving USP inhibitors.

Chemical Structure	Type of USP Inhibitor	Clinical Trials.gov ID	Trial Phase	Study Design	Results	References
**TNG348** 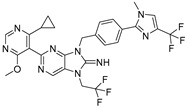	USP1 IC_50_: 82 nM	NCT06065059	Phase 1 dose escalation and Phase 2 dose expansion trial (terminated)	- Target: BRCA 1/2 mutant tumors or HRD+ solid tumors. - Two arms: TNG348 monotherapy and in combination with Olaparib (PARP inhibitor). - Enrollment: 7 patients. - Study period: December 2023–May 2024.	- Terminated due to significant safety concerns. - Grade 3/4 hepatotoxicity observed in patients after 8 weeks. - No patients received combination therapy with Olaparib. - Highlights safety challenges in early-phase trials.	[194,200,201,202]
**KSQ-4279, also known as RO7623066** 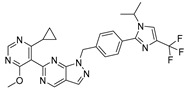	USP1 IC_50_: 10 nM	NCT05240898	Phase 1 (recruiting)	- Target: Advanced solid tumors, particularly those with HRD. - Three arms: monotherapy (arm 1), combination with Olaparib (arm 2), combination with Carboplatin (arm 3). - Dose escalation followed by dose expansion. - Study period: August 2021–June 2027.	- 64 heavily pretreated patients enrolled. - Disease control rates: 28% (monotherapy), 40% (Olaparib combo), 29% (Carboplatin combo). - One partial response in advanced fallopian tube cancer. - 17% (5/29) achieved stable disease for >16 weeks with monotherapy. - Anti-tumor activity in Olaparib combo linked to BRCA1 mutational status. - Most common side effect: anemia (86.7% in Olaparib combo arm). - No study discontinuations due to adverse events. - Study ongoing to determine maximum tolerated dose.	[71,196,203,,204]
**XL309, also known as ISM3091** Chemical structure is not publicly disclosed	USP1	NCT05932862	Phase 1 (recruiting)	- Target: Advanced solid tumors, including those with HRD mutations. - Investigating monotherapy and combination with Olaparib. - Estimated enrollment: 377 patients. - Study Period: August 2023–August 2029.	- No reported results as of December 2024. - Study ongoing.	[197,205,206]
**SIM0501** Chemical structure is not publicly disclosed.	USP1	NCT06331559	Phase 1 (recruiting)	- Target: Advanced solid tumors, including Olaparib-resistant cases. - Estimated enrollment: 176 patients. - Study period: March 2024–December 2027.	- No reported results as of December 2024. - Study ongoing. - First patient enrolled in March 2024.	[197,,207,208]
**HSK39775** Chemical structure is not publicly disclosed.	USP1	NCT06314373	Phase 1 dose escalation and Phase 2 dose expansion trial (recruiting)	- Target: Advanced solid tumors. - Investigating monotherapy in two phases. - Phase 1: Safety, efficacy, and RP2D. - Phase 2: To evaluate ORR and further safety. - Estimated enrollment: 243 patients. - Study period: March 2024–September 2028	- No reported results as of December 2024. - Study ongoing. - First patient enrolled in March 2024.	[197,209,210]
**VLX1570** 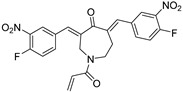	USP14/UCHL5 IC_50_: ~10 µM	NCT02372240	Phase 1 dose escalation and Phase 2 dose expansion trial (terminated)	- Target: Relapsed/refractory multiple myeloma. - Intravenous administration of compound formulated in polyethylene glycol and polysorbate 80 mixture given the poor aqueous solubility. - 28-day cycle with escalating doses. - Enrollment: 14 patients. - Study period: April 2015–May 2017.	- Terminated due to severe toxicity. - 14 patients enrolled. - Anti-myeloma effects observed at doses ≥ 0.6 mg/kg. - Two patient deaths due to abrupt, severe respiratory insufficiency. - Study discontinued due to the severity and abrupt nature of toxicity. - Toxicity comparable to some cases of Bortezomib. - Highlighted the need for further research into safer USP inhibitors.	[198,199,211,212]

## 4. Summary and Future Directions

USP inhibitors have shown significant potential in combating drug resistance and enhancing the effectiveness of existing cancer therapies. The UPS is involved in a vast array of cellular functions; thus, developing more potent and selective USP inhibitors remains a primary focus. Current inhibitors often face challenges related to specificity and potency, given the possible off-target effects these USP inhibitors may have. Researchers are currently exploring novel allosteric binding sites within USPs to design inhibitors with improved selectivity and efficacy. Combination therapies involving USP inhibitors are being studied to overcome drug resistance in cancer treatment. Integrating USP inhibitors with existing anticancer therapies, such as chemotherapy and targeted therapy, shows potential for enhancing treatment outcomes [182]. USP inhibitors also have the key benefit of enhancing the effectiveness of immunotherapy by modulating the tumor microenvironment. Tailored approaches to treatment may lead to more effective and personalized therapy. As research progresses, expanding our understanding of the complex roles of USPs in various signaling pathways and cellular processes will be crucial [54]. Ongoing clinical trials suggest that USP inhibitors, particularly those targeting USP1, may have the potential to become valuable components of cancer therapy. While no USP inhibitors have yet been approved for clinical use, they have shown significant promise in preclinical studies. Advancing USP inhibitors from preclinical studies to clinical trials remains a significant goal, with the aim of bringing these promising therapeutic agents closer to clinical application. As these trials continue to expand and progress, more information about their safety and efficacy in patients will emerge, potentially leading to novel treatment strategies for patients with cancer.

## 5. Conclusions

In conclusion, this review provides an in-depth analysis of USPs’ structural and functional characteristics, their roles in various cancer-related pathways, and the mechanisms of action of over seventy different small molecule USP inhibitors. We also discuss how these inhibitors can enhance cancer immunotherapy, overcome drug resistance, and synergize with existing cancer treatments. Additionally, we explore the current progress and challenges in clinical trials involving USP inhibitors, highlighting their growing potential.

Research on USP inhibitors has shown their potential in targeting oncogenic pathways, modulating immune responses, and mitigating neurodegenerative protein aggregation. Targeting USPs by small-molecule inhibitors has demonstrated significant potential in tackling drug resistance and enhancing the effectiveness of existing cancer therapies. Several USP inhibitors, such as VLX1570 and b-AP15, have demonstrated efficacy in preclinical and early clinical trials, particularly in multiple myeloma and other malignancies.

However, challenges remain in developing selective and potent USP inhibitors due to the highly conserved catalytic domains among DUBs and potential off-target effects. The USPs are involved in multiple pathways, and additional studies are required to understand their complex mechanisms. Further advancements in structure-based drug design, high-throughput screening, and combination therapies may enhance their clinical applicability.

## Figures and Tables

**Table 1:** Cancer-related cellular pathways influenced by USPs.

Pathway	Role of USPs	Cancers	References
NF-κB pathway	Nuclear factor-kappa B (NF-κB) is involved in the regulation of several key biological functions, including cell survival. USPs regulate the NF-κB pathway by modulating the deubiquitination of key signaling proteins. USP21 plays a key role in the downregulation of TNF- α induced NF-κB signaling through deubiquitination of receptor-interacting protein 1 (RIP1), while USP4 promotes TNF-α mediated NF-κB activation by deubiquitinating TGF- β-activated kinase 1, highlighting the diverse roles USPs can play in regulating NF-κB signaling. Downregulation of NF-κB promotes cell death and reduces cell survival.	Breast cancer, prostate cancer, lung cancer, colorectal cancer, and ovarian cancer	[25,26,27]
Wnt/β-catenin pathway	The Wnt/β-catenin pathway is a crucial signaling mechanism that regulates various biological processes, including cell proliferation, differentiation, and survival. β-catenin is a key signal transducer in this pathway and is positively regulated by USP4. The C-terminal catalytic domain of USP4 is responsible for binding and nuclear transport of β-catenin leading to increased transcription of oncogenes. USP4 knockdown in a cell line of colon cancer has also been found to reduce invasion and migration, while overexpression has been demonstrated to enhance β-catenin-regulated transcription.	Colorectal cancer, gastric cancer, breast cancer, lung cancer, pancreatic cancer, and melanoma	[28,29]
JAK/STAT pathway	USPs regulate the JAK/STAT pathway by modulating the stability and degradation of key signaling components, such as STAT3 which plays a crucial role in transcription. Aberrant activation of STAT3 can enhance cancer cell proliferation, increase cell survival by up-regulating anti-apoptotic genes, and may contribute to drug resistance as well as immune evasion. USP5 plays a significant role in the progression and metastasis of pancreatic cancer by stabilizing STAT3.	Breast cancer, lung cancer, colorectal cancer, gastric cancer, leukemia, and bladder cancer	[30,31,32]
p53 signaling pathway	Several USPs regulate the p53 signaling pathway. Importantly, USP7 has been demonstrated to regulate p53 by stabilizing MDM2, an E3 ubiquitin ligase that typically promotes p53 degradation. USP7 has been proposed to be a potential therapeutic target given the contribution it has to cancer pathogenesis through the downregulation of p53. This in turn allows damaged cells to evade apoptosis and proliferate abnormally.	Breast cancer, colorectal cancer, ovarian cancer, lung cancer, and head and neck cancer	[33,34]
c-MYC	c-MYC is a well-established oncogene that plays a critical role in the progression of various cancers. This pathway is significantly influenced by various USPs, such as USP16 and USP36, which interact directly with c-MYC by deubiquitinating and stabilizing it, promoting chemoresistance. USP1 has been demonstrated to promote c-MYC pathway activation, leading to increased transcription of MYC target genes. USP7 indirectly affects MYC levels by stabilizing MDM2 and MDMX which regulate p53 that functions as a MYC antagonist.	Colorectal cancer, breast cancer, non-small cell lung cancer, pancreatic ductal adenocarcinoma, glioblastoma, and ovarian cancer	[35,36,37]
TGF-beta pathway	TGF-beta signaling has been shown to drive epithelial-mesenchymal transition enhancing tumor migration and invasion through pathways regulated by USPs. USP22 is upregulated in many types of cancers. In epithelial ovarian cancer, higher USP22 levels are associated with higher stage and worse clinical outcomes. USP22 facilitates cell proliferation by inducing G1 cell cycle arrest through interaction with oncogenic TGF-β1.	Breast cancer, colorectal cancer, liver cancer, non-small cell lung cancer, pancreatic cancer, and epithelial ovarian cancer	[38,39,40]
DNA repair pathways	USP21 has shown positive regulatory effects on BRCA2 which plays a critical role in homologous recombination. USP1 also plays a critical role in DNA damage repair by modulating the ubiquitination of key regulators of DNA repair such as PCNA and FANCD2.	Breast cancer, non-small cell lung cancer, colorectal cancer, gastric cancer, ovarian cancer, and hepatocellular carcinoma	[3,41,42,43]
Apoptotic pathways	Apoptotic signaling pathways are regulated by USPs. BCL-2 family proteins regulate apoptosis by controlling the release of apoptogenic factors, with both pro-apoptotic and anti-apoptotic proteins regulated by this group of proteins. Overexpression of USP9x in certain cancers leads to the overexpression of Mcl-1, an anti-apoptotic protein, by way of deubiquitination. This has been shown to lead to increased resistance to radiation therapy.	Breast cancer, lung cancer, colorectal cancer, ovarian cancer, and hepatocellular carcinoma	[44,45,46]
Cell cycle regulation pathways	The cell cycle is characterized by stages G1, S, G2, and M. Cyclins, cyclin-dependent kinases (CDKs), cyclin-dependent kinase inhibitors (CDKIs), and aurora kinases function to regulate the cellular transition among these stages. USPs regulate cell cycle progression by stabilizing regulatory proteins such as CDKs. USP2, USP5, USP13, USP20, and USP22 have been found to promote the stability of cyclin D1 which facilitates cell cycle advancement and tumorigenesis.	Breast cancer, lung cancer, colorectal cancer, gastric cancer, and prostate cancer	[16,44,47,48,49]
MAPK pathway	The MAPK (mitogen-activated protein kinase) pathway is a system of intracellular communication that transmits signals from the cell surface to the nucleus. USP11 promotes the progression and chemoresistance of various cancers, and overexpression of USP11 has been associated with abnormal activation of the ERK-dependent MAPK pathway during the progression of colorectal carcinoma.	Breast cancer, colorectal cancer, lung cancer, gastric cancer, ovarian cancer, and hepatocellular carcinoma	[44,50]
Hypoxia pathways	Hypoxic conditions in tissues, particularly in cancer, are known to induce a cellular transcriptional response. Transcriptional complex hypoxia-inducible factors (HIF) are the key factors in this signaling pathway and the α-subunit of HIF triggers a cascade that leads to degradation by the 26S proteasome. USP22 has been shown to regulate the hypoxia pathway by stabilizing HIFs such as HIF-1α and enhancing their transcriptional activity, allowing cancer cells to adapt to hypoxic conditions.	Breast cancer, lung cancer, colorectal cancer, pancreatic cancer, cervical cancer, and head and neck cancer	[51,52]

**Table 3:** Small molecule inhibitors targeting USPs.

USP Target	Name of the Inhibitor	Chemical Structure	IC_50_ Values	Mechanism of Action and Anticancer Activity	Refs
USP1	SP-002	Structure not disclosed.	15.7 nM	- Highly selective USP1 inhibitor. - Induces accumulation of Ub-PCNA on chromatin leading to S/G2 cell cycle arrest. - Synergistic with the PARP inhibitor olaparib in malignancies with homologous recombination defects (HRD). - Minimal side effects on hematopoietic stem cells in vitro. - Tumor growth suppressed in in vivo xenograft mice model.	[71,72]
USP1 + UAF1	C527	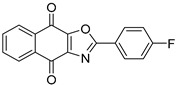	0.88 µM	- A non-covalent inhibitor that binds reversibly to the active site of the USP1/UAF1 complex. - Promotes ID1 degradation which is a transcription factor essential for the proliferation of many types of cancer as well as upregulates cell cycle inhibitor p21. - Enhances cisplatin sensitivity in vitro and in vivo.	[73,74]
	SJB2-043	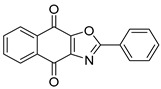	0.544 µM	- More potent derivative of C527. - Reversibly binds to and inhibits the enzymatic activity of the USP1/UAF1. - Leads to degradation of ID1/ID2/ID3 and apoptosis. - Inhibits growth of myeloid leukemia and multiple myeloma cells.	[73,75]
	SJB3-019A	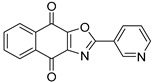	78.1 nM	- Similar mechanism of action as SJB2-043 but more potent. - Increases the level of Ub-FANCD2 and Ub-PCNA and leads to inhibition of DNA repair by decreasing homologous recombination activity.	
	ML323	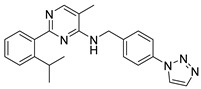	76 nM	- Allosterically leads to changes of the active site and disrupts the hydrophobic core of USP1. - Inhibits PCNA and FANCI-FANCD2 deubiquitination, leading to disruption of DNA replication and repair pathways in cancer cells.	[76,77,78]
USP1 + UAF1/USP7	Pimozide	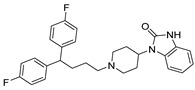	2 µM/47 µM	- A non-covalent and non-competitive inhibitor that binds to the USP1/UAF1 complex and reversibly inhibits its enzymatic activity. - Upregulates cell cycle inhibitory proteins p21 and p27 as well as downregulates cell cycle promoting proteins cyclin D3 and CDK2. - Induces ubiquitination and degradation of Max (a MYC binding protein). - Leads to G0/G1 arrest and apoptosis in diffuse large B-cell lymphoma (DLBCL). - Reduces tumor burden in rituximab-resistant DLBCL cells- and patient-derived xenograft (PDX) mouse models. - Shown to inhibit breast cancer and non-small cell lung cancer (NSCLC) cell proliferation.	[79,80,81]
	Trifluoperazine (TFP)	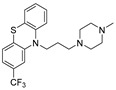	8 µM/9 µM	- A non-covalent inhibitor that binds reversibly to the USP1/UAF1 complex and inhibits its activity. - Classified as a typical antipsychotic agent. - Decreases cyclin D1/CDK4 and cyclin E/CDK2 which stimulates intrinsic apoptosis. - Leads to G0/G1 cell cycle arrest and apoptosis in triple-negative breast cancer (TNBC). - Inhibits TNBC tumor growth.	[79,82]
	Flupenthixol	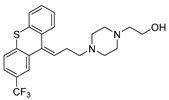	7 µM/13 µM	- A non-covalent non-competitive inhibitor that binds reversibly to the USP1/UAF1 complex and inhibits its activity. - Similar in structure to TFP and classified as a typical antipsychotic medication. Less selective than Pimozide and GW7647.	[79]
USP1 + UAF1/USP2/USP7/USP8	Rottlerin	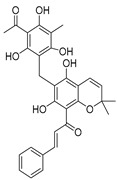	8 µM/34 µM/13 µM/6 µM	- Irreversibly inhibits the USP1/UAF1 complex. - Contains an α, β-unsaturated carbonyl group, which acts as a potential Michael acceptor. - Covalently modifies the cysteines at the active sites. - Causes G0/G1 cell cycle arrest. - Downregulates cyclin D1. - Suppresses NF-kB and its target genes. - Induces antiangiogenic effects and causes apoptosis in breast cancer cells.	[79,83,84]
USP1 + UAF1/USP12	GW7647	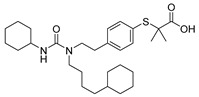	5 µM/44 µM	- A potent non-competitive reversible inhibitor of the USP1/UAF1 complex. - Binds to an allosteric site. - May sensitize cells to DNA-damaging agents as the timely deubiquitination of PCNA and FANCD2 is important for proper DNA repair. - Enhances the cytotoxicity of cisplatin in NSCLC.	[79,85]
USP 1/2/3/4/5/7/8/15/20/28/47/UCHL1/UCHL3/UCHL5	PR619(Broad range or Pan DUBs inhibitor)	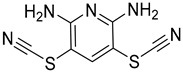	3.9–8.9 µM	- Pan inhibitor of multiple USPs. - Binds reversibly to the active sites of several USPs and interferes with the removal of ubiquitin from the respective substrate. - Triggers apoptosis by activating caspases and PARP cleavage which leads to G0/G1 cell cycle arrest. - In vivo xenograft mouse model demonstrated inhibition of human chondrosarcoma tumor growth.	[54,86,87]
USP2	Q29	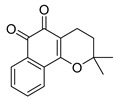	<5 µM	- Selective, irreversible inhibitor of USP2. - Causes oxidation of the catalytic cysteine residue of USP2. - Interferes with cancer cell cycle progression.	[88,89]
	ML364	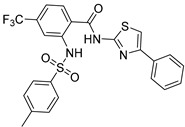	1.1 µM	- Reversibly and non-competitively inhibits USP2 at an allosteric site. - Interrupts cyclinD1-USP2 interaction. - Enhances cyclinD1 degradation. - Causes G0/G1 cell cycle arrest in colorectal cancer cells. - Decreases homologous recombination-mediated DNA repair.	[89,90]
	LCAHA	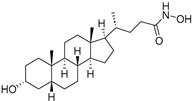	3.7–9.7 µM	- Selective, non-competitive inhibitor. - Destabilizes cyclin D1. - Causes G0/G1 cell cycle arrest in colon cancer cells.	[89,91]
	STD1T	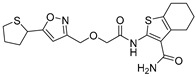	3.3 µM	- Selective, reversible inhibitor of USP2a. - Inhibits deubiquitination of cyclin D1. - Leads to G1 phase cell cycle arrest. - Enhances the cyclin D1degradation in colon cancer and breast cancer cells.	[89,92]
	6TG	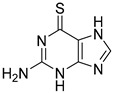	40 µM	- Non-competitive, slow-binding covalent inhibitor of the USP2 catalytic domain. - Disrupts enzyme–substrate interaction and covalently binds to the Cys276 of USP2.	[89,93,94]
USP2/5/8/UCHL1/UCHL3	RA-9	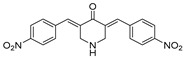	Not determined by enzymatic assay	- Non-specific irreversible inhibitor - Exposes its carbonyl to nucleophilic attack by cysteine - Downregulates cyclin D1, upregulates p53, p27Kip1, and p16Ink4A. - Causes G2-M phase cell cycle arrest and apoptosis. - Increases ER stress markers. - Reduces tumor growth and prolongs survival in ovarian cancer xenograft mouse model.	[54,89,95,96,97]
	RA-14	Chalcone derivative of RA-9 (structure not disclosed)	Not determined by enzymatic assay	- Derivative of RA-9 - Irreversible USP inhibitor. - Presence of the α, β-unsaturated carbonyl group of RA-14 is susceptible to nucleophilic attack from the sulfhydryl group of the USP catalytic cysteine residues - Causes S-G2/M phase cell cycle arrest. - Downregulates cyclinD1, upregulates p53, p27Kip1, and p16Ink4A. - Induces apoptosis in breast, cervical, and ovarian cancer cells.	[54,95]
	AM146	Chalcone derivative of RA-9 (structure not disclosed)	Not determined by enzymatic assay	- Derivative of RA-9. - Similar mechanism of action as RA-9 - Downregulates cyclin D1. - Upregulates p53.	[54,95]
USP2/7	NSC632839	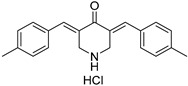	45 μM/37 μM	- Inhibits cleavage of z-LRGG sequence of Ub of USP. - Non-selective USP2 and USP7 isopeptidase inhibitor - Also inhibits deSUMOylase SUMO specific protease 2 (SENP2). - Induces apoptosis by stabilizing the second mitochondrial-derived activator of caspases	[89,98]
	Compound 14	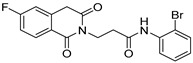	0.25 µM (USP2)	- Reversible inhibitor of USP2 and USP7. - Uncompetitive mechanism of action.	[54,89,99]
USP4/5	Vialinin A	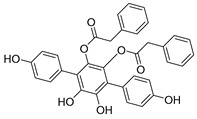	1.5 µM/5.9 µM	- Non-covalent inhibitor. - Binds specifically, to the catalytic domain near Val98 within USP4 and forms H-bonds via residues Asp91, Glu92, and Leu97. - Forms H-bonds via residues Asp91, Glu92, and Leu97 on the USP4. - π-stacking interactions with residues Phe44 and Phe53 contribute to stability. - Isolated from the Chinese mushroom Thelephora vialis. - Inhibits TNF-α translation and enhances its degradation. - Inhibits TGF-β induced cell proliferation.	[100,101,102]
USP5	USP5-IN-1 (compound 64)	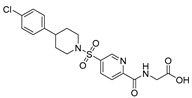	0.8–26 µM	- Non-covalent inhibitor. - Selective inhibitor of USP5. - Binds to the C-terminal ubiquitin-binding site of the ZnF-UBP domain. - Displaces ubiquitin in a concentration-dependent manner.	[103]
USP5/7/8/13/14/15/22	Curcusone D	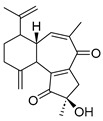	Not determined by enzymatic assay	- Covalent, non-selective inhibitor of USPs. - Induces ROS production. - Inhibits cancer cell growth in combination with Bortezomib in multiple myeloma cells.	[54,104]
USP5/9X/14/24/37/UCHL5	WP1130 (Degrasyn)	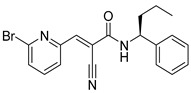	2.5–5 µM	- Partly selective non-covalent USP inhibitor. - Inhibition of USP9X leads to downregulation of survivin, c-FLIP, and MCL-1 (anti-apoptotic proteins). - Sensitizes cancer cells to TNF-related apoptosis-inducing ligand mediated apoptosis. - Increases sensitivity of NSCLC cells to cisplatin	[100,105,106,107,108]
	EOAI3402143 (G9)	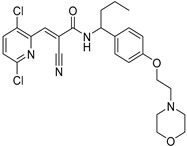	1.6–5 µM	- Reversibly targets multiple USPs. - Downregulates cyclin D1. - Suppresses NSCLC proliferation in a xenograft mouse model.	[49,107,109,110]
USP7	HBX-41108	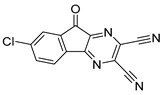	0.424 µM	- Reversible and uncompetitive USP7 inhibitor. - Upregulates p53 and p21. - Induces apoptosis in cancer cells. - Antiproliferative against colon cancer cell line.	[111,112]
	HBX-28258	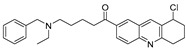	22.6 µM	- Covalent inhibitor that binds to the active site of USP7 at Cys223Promotes MDM2 degradation and p53 activation in colon cancer cells.	[113,114]
	Spongiacidin C	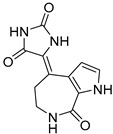	3.8 µM	- Selective USP7 inhibitor - One of the first USP7 inhibitors derived from a natural source. - Pyrrole alkaloid isolated from the marine sponge Stylissa massa. - Inhibition of USP7 leads to MDM2 and MDMX degradation which in turn promotes p53 upregulation. - In vitro studies demonstrated a lack of cytotoxic activity.	[114,115]
	GNE-6640	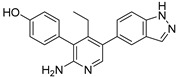	0.75 µM	- Bind to the ubiquitin-binding site within the USP catalytic domain. - Interacts with the acidic residues that mediate hydrogen-bond interactions with the ubiquitin Lys48 side chain. - Increases ubiquitination and degradation of MDM2, resulting in stabilization of p53.	[116,117,118]
	GNE-6776	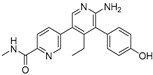	1.34 µM		
	XL188	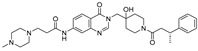	90 nM	- Targets the S4-S5 pocket of catalytic domain ~5 angstroms from the cysteine residue. - The 4-hydroxy-piperidine group of XL188 forms hydrogen bonds with the Gln297 carboxylic group of USP7. - Leads to increased expression of tumor suppressors p21 and p53.	[119,120]
	ALM4	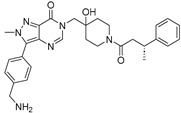	6 nM	- Both ALM4 and ALM46 are highly potent non-covalent inhibitors. - Binds to the USP7 catalytic domain. - Promotes MDM2 and MDMX degradation, which in turn activates p53.	[121,122]
	ALM46	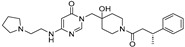	0.09 µM		
	FT671	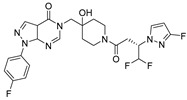	52 nM	- High affinity and specific non-covalent inhibitor. - Targets dynamic pocket near the catalytic center. - Downregulates USP7 substrates (e.g., MDM2). - Increases expression of tumor suppressors p53 and p21. - Reduces TNBC tumor growth in vivo.	[123,124]
	FT827	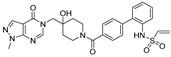	*K*_i_ = 66 µM	- Covalent inhibitor of USP7. - The vinylsulfonamide moiety covalently binds at Cys223. - Specifically targets USP7 at the active site. - Similar downstream effects of FT671.	[123]
	Parthenolide (PTL)	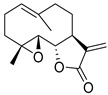	6.58 µM	- Covalent inhibitor. - Contains an α-methylene-γ-butyrolactone moiety, which directly interacts with USP7 in a competitive manner. - Covalently modifies the Cys223 residue. - Induces G2/M phase cell cycle arrest and apoptosis. - Disrupts wnt/β-catenin signaling pathway by enhancing β-catenin ubiquitination and degradation. - Suppresses colorectal cancer cell proliferation in vitro. - Leads to the inhibition of the NF-kB pathway. - Stabilizes p53 by promoting MDM2 ubiquitination and degradation. - Suppresses JAK activity reducing STAT3 activation. - Reduces tumor burden in colon cancer xenograft mouse model.	[125]
	Costunolide	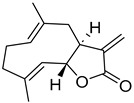	Not determined by enzymatic assay	- Covalent inhibitor. - Contains an α-methylene-γ-butyrolactone moiety, which directly interacts with USP7. - Degradation of β-catenin leads to suppression of the Wnt/β-catenin signaling pathway. - Inhibits growth of colon cancer cells in vitro.	[125]
	YCH2823	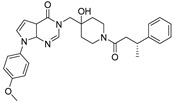	49.6 nM	- Directly interacts with the catalytic domain. - Impedes deubiquitination of substrates such as MDM2. - Increases expression of tumor suppressors p53 and p21 levels. - Causes G1 phase cell cycle arrest.	[126]
	XL177A	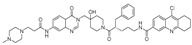	0.34 nM	- Covalent, irreversible, and extremely potent sub-nanomolar inhibitor. - Irreversibly binds to USP7 by forming a covalent bond with the catalytic cysteine. - Induces conformational changes in USP7 protein dynamics. - Degradation of negative regulators of p53 (e.g. MDM2), which leads to G1 cell cycle arrest. - Exerts anti-tumor activity against Ewing sarcoma and malignant rhabdoid tumor.	[120]
USP7/10	HBX-19818	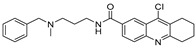	28.1 µM/14 µM/	- Covalent inhibitor. - Irreversibly targets the USP7 catalytic site at Cys223 through nucleophilic attack, resulting in chloride release from the molecule and subsequently covalent binding. - Promotes MDM2 protein degradation and p53 activation. - Induces caspase 3 activity and PARP cleavage. - Induces G1 phase cell cycle arrest. - Reduces colon cancer cell proliferation.	[113,114,127]
	Compound 3	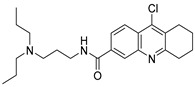	3.6 µM (USP7)	- Non-covalent inhibitor that makes H-bond with Asp295 and hydrophobic contact with Phe409 in the binding pocket. - Structural analog of HBX-19818. - More specific to USP10 than USP7. - Leads to FMS-like tyrosine kinase 3 (FLT3) degradation at lower concentrations.	[54,62,127,128]
USP7/47	P22077	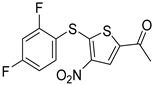	8 µM (USP7)	- Covalent inhibitor. - Binds to the Cys223 residue at the catalytic site which induces the confirmational change in active site rearrangement to inhibit enzymatic activity. - Disrupts the NF-kB and MAPK pathways. - Stabilizes p53 by triggering HDM2 degradation. - Re-sensitizes chemo-resistant neuroblastoma cells. - Reduces tumor burden in orthotopic neuroblastoma xenograft mouse model. - Overcomes cytarabine resistance in AML cells.	[54,86,129,130,131,132]
	P5091	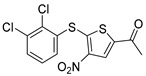	4.2 µM (USP7)	- Selective and potent covalent inhibitor - Enhances β-catenin degradation, which leads to disruption of the wnt/β-catenin signaling pathway and induces apoptosis. - Suppresses colorectal cancer cell proliferation in vitro and in vivo. - Induces apoptosis in multiple myeloma cells that are resistant to Bortezomib.	[54,133,134,135]
	P50429	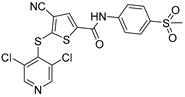	0.42 µM/1 µM	- Covalent analog of P5091. - Covalently modifies the catalytic cysteine residue, Cys223, at the active site of USP7. - Induces conformational switch in USP7 active site. - Inhibits colon cancer cell proliferation.	[54,132,136]
USP8	HY50736	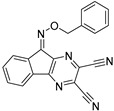	0.24 µM	- Mainly target USP8 with very poor activity against USP7. - Derivatives of HBX-41108. - The introduction of the O-alkyloxime moieties at C-9 of the tricyclic scaffold is important for USP8 inhibition. USP8 plays a key role in the recycling of cell surface receptors such as EGFR, and inhibition leads to decreased cellular survival and proliferation.	[54,137]
	HY50737A	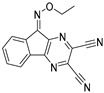	0.28 µM		
USP9x	FT709	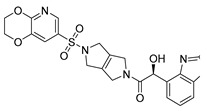	82 nM	- Non-covalent, potent, and highly selective inhibitor. - Competitive USP9x inhibitor. - Destabilizes Makorin and ZNF598 ubiquitin E3 ligases, which are substrates that regulate the ribosomal quality control pathway.	[138]
USP10	Compound 9	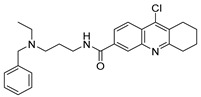	Not determined by enzymatic assay	- Non-covalent inhibitor. - Interacts with Asp295, Val296, and Tyr456 at the catalytic domain. - Structural analog of HBX-19818. - Similar in structure to compound 3 but lacks activity against USP7. - Reversible inhibition of USP10 leads to FLT3 degradation.	[54,62,127,128]
	Wu-5	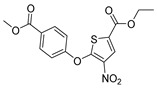	8.3 µM	- Selective covalent USP10 inhibitor. - Induces degradation of FLT3-ITD and shows efficacy against FLT3 inhibitor-resistant AML cells. - Enhances anti-leukemic effects of FLT3 inhibitors (crenolanib).	[139,140]
USP10/13	Spautin-1	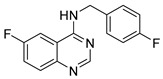	0.6–0.7 µM	- Potent and specific inhibitor. - Leads to beclin-1 degradation, which is important for autophagy. - Reduces cancer cell proliferation in nutrition-deprived conditions. - Prevents induction of UPR-associated proteins and induces cytotoxicity preferentially in glucose-starved cancer cells.	[141,142,143]
USP11	Mitoxantrone	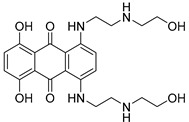	3.15 µM	- Synthetic anthracenedione. - Inhibits the activity of USP11, however, the exact mechanism of action is elusive. - USP11 plays an essential role in DNA repair. - Exhibits anticancer activity through several mechanisms, such as DNA intercalation to produce DNA strand breaks, topoisomerase II inhibition, and interference with RNA synthesis.	[144]
USP12/USP46	Galeterone	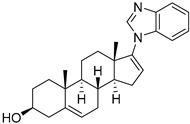	2.1–3.4/3.4–4.2 µM	- Selectively inhibits USP12 and USP46. - Disrupts androgen receptor (AR) stability and the signaling pathway. - Suppresses prostate cancer cell proliferation in vitro.	[145]
USP14	IU1	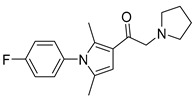	4–5 µM	- Reversible and selective USP14 inhibitor. - Promotes MDM2 protein degradation in cervical cancer cells leading to G0/G1 cell cycle arrest.	[146,147]
	IU1-47	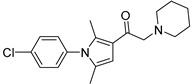	0.6 µM	- More potent derivatives of IU1 with a similar mechanism of action, but several folds more potent. - Prevents USP14 from docking on the proteasome. - Binds to the thumb-palm cleft region of the catalytic domain of USP14 which prevents the C-terminus of ubiquitin from accessing USP14. - Promotion of proteasome substrate degradation of proteins crucial for cancer cell survival. - IU1-248 shows evidence of synergy with the PARG inhibitor COH34 against TNBC in vitro.	[146,148,149,150]
	IU1-206	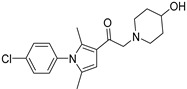	Not determined by enzymatic assay		
	IU1-248	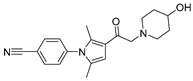	0.83 µM		
USP14/UCHL5	Auranofin	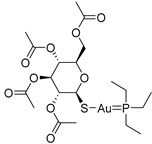	Not determined by enzymatic assay	- Competitively reduces substrate binding (HA-Ub-VS) to the active site Cys of USP14. - FDA approved gold containing disease-modifying antirheumatic drug (DMARD).	[151,152]
	CuPT	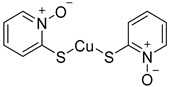	Not determined by enzymatic assay	- These inhibitors compete with Ub-VS for binding to UCHL5 and USP14. - Metal-center containing compounds with an identical organic structure but different metal. - Induce G2 cell cycle arrest. - Lead to accumulation of ubiquitinated proteins inducing G2 cell cycle arrest.	[153,154,155,156,157]
	PtPT	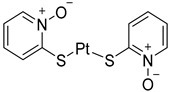	Not determined by enzymatic assay		
	NiPT	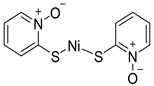	Not determined by enzymatic assay		
	b-AP15	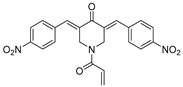	<10 µM	- Covalent inhibitor. - Selectively inhibits the active site at residues Cys203 and Cys257 in the ubiquitin-binding pocket of USP14. - Contains an α, β-Unsaturated carbonyl - Contains electrophilic Michael acceptor motifs which contribute to its ability to form covalent bonds with its targets via Michael addition. - Reaction forms high molecular weight complexes, which can be reduced by thiol-containing reducing agents such as dithiothreitol (DTT) or glutathione. - Alters levels of cell cycle regulators such as cyclin D1, CDKs, and p27 which leads to G0/G1 cell cycle arrest and apoptosis.	[158,159,160,161]
USP22	USP22i-S02	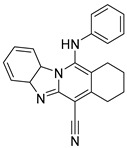	Not determined by enzymatic assay	- Potent, reversible USP22 inhibitor. - Leads to the degradation of cell cycle progression proteins including cyclin B1 and cyclin D1. - Synergizes with cisplatin in breast cancer cells. - Suppresses Foxp3 in T regulatory cells (Tregs).	[162,163,164,165]
USP24	NCI677397	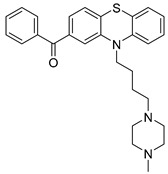	Not determined by enzymatic assay	- Non-covalent inhibitor. - Interacts with the catalytic domain of USP24. - Leads to decreased stability of the ATP-binding cassette (ABC) transporters which function to pump toxins including chemotherapy out of cells. - Potential ferroptosis inducer (FIN) by increasing levels of lipid ROS leading to ferroptotic cellular death.	[118,166]
	USP24-i-101	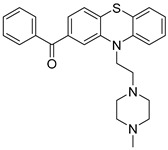	Not determined by enzymatic assay	- Reversible inhibitor. - Increases PD-L1 degradation leading to increased apoptotic signaling. - Activates autophagy by inhibiting E2F4 and TRAF6. - Excessive activation of autophagy paradoxically leads to overcoming chemotherapy resistance in drug-resistant lung cancer cells in vitro, as excessive autophagy induction can lead to cell death when combined with chemotherapy. - Overcomes Taxol- or gefitinib-induced drug resistance in lung cancer.	[167]
USP25/28	AZ1	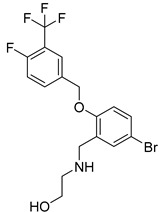	1.08 µM/1.76 µM	- Non-competitive dual inhibitor. - Benzylaminoethanol derivative. - Binds to the conserved site of USP25 and USP28 at the distal portion of the S1 cleft. - Fluorine is essential for binding and allosteric regulatory inhibition. - Several USP25 and USP28 substrates function as oncogenes such as ΔNp63.	[168,169]
	FT206	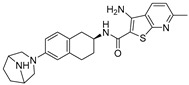	1.01/0.15 μM	- Binds to the same cleft as AZ1. - Higher selectivity for USP28. - Destabilizes c-MYC. - Induces regression of squamous cell lung cancer in vivo.	[37,169]
	Vismodegib (VSM)	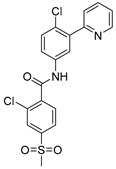	2.92/3.51–4.41 μM	- Interacts with the binding pocket of USP28 composed of two helical structures spanning D255-N278 and N286-Y293. - Leads to downregulation of c-MYC, Notch1, and Tankyrase-1/2 in colorectal cancer cells. - Inhibits the Hedgehog signaling pathway, which has been implicated in the pathogenesis of basal cell carcinoma.	[169,170,171,172]
USP30	MF-094	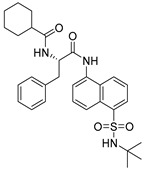	0.12 µM	- Selectively inhibits the active site of USP30, a mitochondrial localized DUB. - Enhances mitophagy by suppressing deubiquitination and enhancing the degradation of damaged mitochondria. - Inhibits oral squamous cell carcinoma in vivo.	[173,174,175]
	15-oxospiramilactone	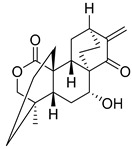	Not determined by enzymatic assay	- Binds to Cys77 of the catalytic domain of USP30. - Semi-synthetic product isolated from the Spiraea japonica plant. - Promotes mitochondrial fusion by enhancing the activity of mitofusin proteins Mfn1 and Mfn2.	[176,177]
	Compound 39 (CMPD-39)	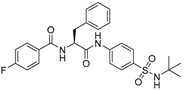	20 nM	- Selective competitive inhibitor. - Binds to the catalytic site of USP30. - Increases ubiquitination of TOMM20 and SYNJ2BP, which leads to increased mitophagy.	[178,179,180]
USP36	Cinobufotalin	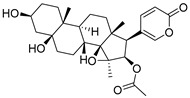	2.75 μM	- Interacts directly with the catalytic domain of USP36. - Is a type of bufadienolide, which is a subclass of cardiac glycosides that are derived from toad venom glands. - Promotes the degradation of c-Myc. - Inhibits proliferation, migration, and invasion of colon cancer cells.	[36]

## Data Availability

Not applicable.
